# Micromechanical Models for FDM 3D-Printed Polymers: A Review

**DOI:** 10.3390/polym15234497

**Published:** 2023-11-23

**Authors:** Rowin J. M. Bol, Branko Šavija

**Affiliations:** Microlab, Faculty of Civil Engineering and Geosciences, Delft University of Technology, Stevinweg 1, 2628 CN Delft, The Netherlands; b.savija@tudelft.nl

**Keywords:** fused deposition modeling (FDM), additive manufacturing (AM), printing process parameters, mechanical anisotropy, inter-layer bond, intra-layer bond

## Abstract

Due to its large number of advantages compared to traditional subtractive manufacturing techniques, additive manufacturing (AM) has gained increasing attention and popularity. Among the most common AM techniques is fused filament fabrication (FFF), usually referred to by its trademarked name: fused deposition modeling (FDM). This is the most efficient technique for manufacturing physical three-dimensional thermoplastics, such that FDM machines are nowadays the most common. Regardless of the 3D-printing methodology, AM techniques involve layer-by-layer deposition. Generally, this layer-wise process introduces anisotropy into the produced parts. The manufacturing procedure creates parts possessing heterogeneities at the micro (usually up to 1 mm) and meso (mm to cm) length scales, such as voids and pores, whose size, shape, and spatial distribution are mainly influenced by the so-called *printing process parameters*. Therefore, it is crucial to investigate their influence on the mechanical properties of FDM 3D-printed parts. This review starts with the identification of the printing process parameters that are considered to affect the micromechanical composition of FDM 3D-printed polymers. In what follows, their (negative) influence is attributed to characteristic mechanical properties. The remainder of this work reviews the state of the art in geometrical, numerical, and experimental analyses of FDM-printed parts. Finally, conclusions are drawn for each of the aforementioned analyses in view of microstructural modeling.

## 1. Introduction

Additive manufacturing (AM) and rapid prototyping (RP) are popular names associated with 3D-printing techniques [1]. The principle behind the fabrication of these 3D-printing methods is to slice three-dimensional objects from virtual models, obtained through computer-aided design (CAD) software [2], and build them layer by layer [3]. Due to its large number of advantages compared to traditional subtractive manufacturing techniques [4], such as its cost-efficiency [1,5,6], prototyping speed [1,7,8,9,10], high flexibility in producing complex geometries [1,4,5,6,7,9,11,12], reduced material waste [1,5,10], decreased time and labour costs [13], and facilitation of product customization [3,10,12], this process has gained increasing attention [11] and popularity [14]. Therefore, 3D-printing technology applications can be found in a large variety of sectors, not limited to the automotive [9], aerospace engineering [5,9,15], medical application [9], biomedical engineering, civil engineering [15], marine engineering [16], clothes, and music [17] industries. However, the disadvantages are that these processes are subject to problems of accuracy [10,18]; difficulty in performance tailoring [10] (defects, durability, and anisotropy in particular [3,8,10,14,19]); poor surface quality [20,21] and roughness [10,22,23]; low production rates; a lack of regulation and standards; and the limited size of parts [10]. Hence, it is important to develop a profound understanding of 3D-printed polymers’ microstructural mechanical characteristics before these materials are allowed to be used in final consumer products [24,25].

Compared to metal and metallic material printing, the 3D printing of polymers is much more accessible and economical considering the printing equipment requirements, raw material production, and printing costs [26]. Among the most common AM techniques is material extrusion [27], also known as fused filament fabrication (FFF), the generic name for extrusion-based processes in which molten extrudate is deposited through a nozzle with a small diameter ranging from about 0.1 mm to 1 mm [28], usually referred to by its trademarked name: fused deposition modeling™ (FDM) [19]. Stratasys Inc., Eden Prairie, MN, USA, developed FDM [8] in 1990 [1,13]. The FDM technique essentially consists of three stages: (i) design generation, (ii) machine code (G-code) generation, and (iii) fabrication [9]. The process is initiated through the creation of a three-dimensional solid model, which can be carried out with many sophisticated CAD software options [13,18]; this is stage (i). In stage (ii), the model is converted into 3D stereolithography format, i.e., standard triangulation language (STL) file format [1,13,18,22]. This format tessellates the model into a set of triangles, after which it is sliced horizontally into a large number of thin sections [18]. These slices depict two-dimensional contours, which altogether form the tool paths for each layer to be deposited. This set of instructions is called the G-code, which is produced by devoted software, usually referred to as *slicer* or *slicing software* [2], and contains all information about the filament supply, nozzle and base plate temperatures, and extrusion head and platform motions for extruding the fused filament. Finally, in step (iii), the specific printing instructions in the form of the G-code are sent to the FDM machine for fabrication [9].

**Figure 1:**
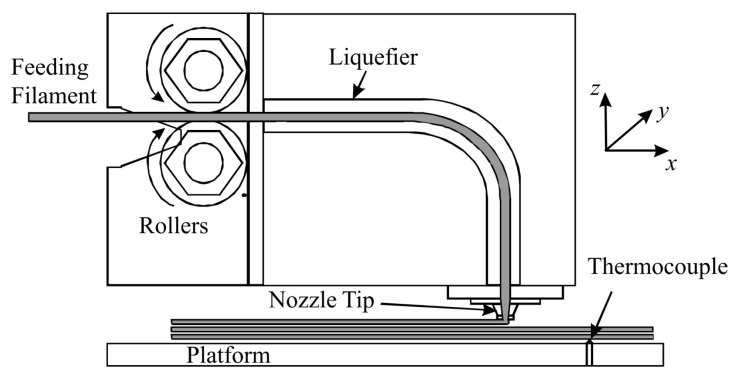
FDM 3D-printing technique [12]. Reproduced with permission from Emerald Publishing Limited, 2008.

Besides being one of the most widely adopted AM and RP technologies [1,6,19,21,24], FDM is the most efficient [22] technique for manufacturing physical three-dimensional thermoplastics [7]. As a result, FDM machines are nowadays the most common [21]. During the FDM process, a thermoplastic filament is heated and extruded in a prescribed layer-by-layer pattern onto a platform [2,7,12,22]; see Figure 1. Each printed layer is composed of filaments, also known as fibers, beads, or roads [2]. Since the thermoplastic filament is extruded and deposited in a semi-molten state, the added material fuses with the already deposited neighboring filaments, after which it cools down, solidifies, and bonds with the adjoining material [8,18]. In the case of traditional Cartesian 3D printers, after finishing a layer, the platform (see Figure 1) moves downwards by the height of one filament and continues with the deposition of the next layer. On the other hand, more modern Delta 3D printers rely on a fixed platform, whilst the extrusion head changes height.

In general, various types of thermoplastics can be used in the FDM process [1,27]. The material most commonly used is acrylonitrile butadiene styrene (ABS) [2,3,5,6,8,9,10,11,12,13,18,19,24,28,29,30,31,32,33,34,35,36], followed by poly-lactic acid (PLA) [4,7,9,14,15,24,25,37,38,39,40,41]. Other than these, widely used FDM filaments include thermoplastic polyurethane (TPU) [1,10]; polycarbonate (PC) [1,9]; polyetherimide (PEI) [9]; polyaryletherketone (PAEK) [28]; and polyetheretherketone (PEEK) [9], which has particularly high strength and thermal resistance. The micromechanical models for FDM 3D-printed polymers covered in this review mainly relate to ABS and PLA in view of their regular application [27].

Most of the existing reviews available in the literature mainly focused on a single material or only addressed one of the many aspects of 3D-printed polymers. For example, a review by Khan and Kumar [36] was limited to the printing process parameters in relation to the mechanical characteristics of ABS. Another review by Paul [1] concentrated on finite element analyses (FEA) with only brief descriptions of experimental validation strategies. Other reviews, such as that by Zohdi and Yang [42], discussed a large variety of (pre-)treatments and post-processing techniques with the aim of intercepting the cause of mechanical anisotropy. In view of this, the scope of the present review is to provide an overview applicable to different types of thermoplastics that can be used in the FDM process while dealing with multiple 3D-printing aspects in more depth. This will be accomplished by explaining and emphasizing the significance of the aforementioned printing process parameters, after which the review will delve into their intricate influence on the micromechanical composition of FDM 3D-printed polymers and investigate their subsequent impact on the mechanical properties. A thorough exploration follows, encompassing geometrical, numerical, and experimental analyses related to material extrusion, culminating in insightful conclusions for each of these analytical approaches in the context of microstructural modeling.

## 2. Identification of Printing Process Parameters

Regardless of the 3D-printing methodology, AM techniques involve layer-by-layer deposition [4,9,21,24,25,27]. Generally, this layer-wise process introduces anisotropy in the produced parts, as pointed out by most studies on this subject [3,6,9,13,14,18,19,29,37]. The manufacturing procedure creates parts with heterogeneities at the micro (usually up to 1mm) and meso (mm to cm) length scales, such as voids and pores, whose size, shape, and spatial distribution are mainly influenced by the so-called *printing process parameters* [7,24] or *build parameters* [9]. These geometrical variations result in parts having the same net shape at the macroscale, while their mesostructures can be very different [29]. Most studies have shown that mechanical properties are negatively affected by these parameters [1,4,6,7,8,11,12,13,14,15,22,24,28,29,37,38]. Since FDM is a complex process that involves many process parameters [38], the resulting mechanical properties are often uncertain [8] and can be greatly influenced by small changes in any parameter. The internal composition at the sub-millimeter scale, i.e., the *mesostructure* [2], thus depends on the printing process parameters. Therefore, it is crucial to investigate their influence on the mechanical properties of FDM 3D-printed parts [24,38]. In what follows, the parameters affecting the (mechanical) properties of FDM parts are identified.

A vast amount of research has been performed on the subject of 3D-printing process parameters for FDM, describing their effect on material characteristics and behavior [38]. The studied FDM process parameters include: porosity or solidity [1,7,19,28,38], printing path [1,2,3,4,5,6,8,9,11,13,18,20,22,38], infill degree or air gap [1,2,3,5,6,8,9,11,13,18,22,38,42], feed rate or infill speed [5,9,11,22,38,42], raster orientation [1,2,3,4,6,8,9,11,14,18,19,22,29,38,42], bead width [2,3,4,5,6,8,11,13,18,22,38,42], model temperature [1,2,4,5,9,11,13,18,28,42], nozzle size [11,18], surface roughness and quality [1,11,13,20,21,22], color [13,18], build direction [4,5,6,8,19,21,22,28,38,42], number of contours [2,6,8,9,22], and inclination angle [1,20]. Since the above list of parameters is quite extensive, it can be concluded that it is difficult to study and include all of them in a model. Furthermore, it is not always possible to have complete control over all printing process parameters, e.g., when closed-control FFF printers are used, with which the access to most parameters is restricted [19]. This review only includes process parameters that are considered to affect the micromechanical composition of 3D-printed polymers.

**Figure 2:**
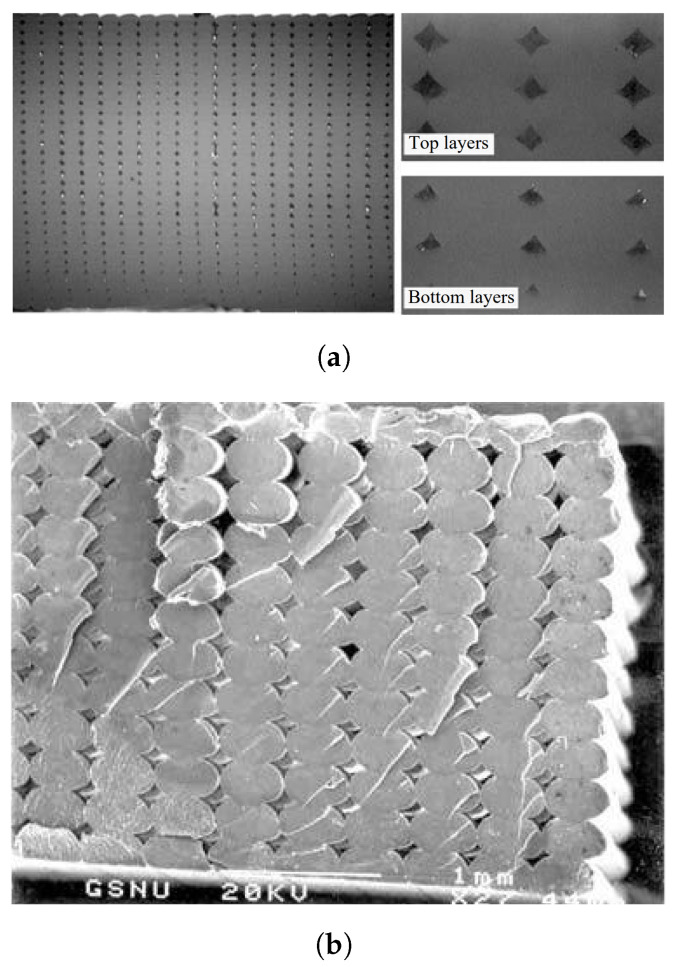
Enlarged section of an FDM-printed part [23]. (**a**) Side view of extruded polymer [12]. (**b**) Shape of deposited filaments [18]. Reproduced with permission from Emerald Publishing Limited, (**a**) 2008, (**b**) 2002.

**Figure 3:**
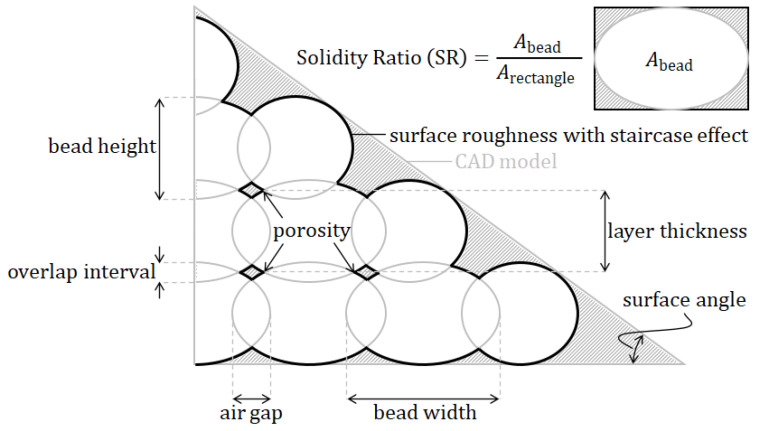
Schematic cross-section explaining several printing process parameters.

Figure 2 depicts a magnified cross-section of an FDM part in which the shape of the deposited filaments resemble elliptic curves [23]. The cross-sectional shape parameters of these filaments, often referred to as beads, are therefore considered to have a major effect on the micromechanical characteristics of the 3D-printed material. The cross-sectional printing process parameters taken into account in this study are the *bead width* and *layer thickness*. The bead width is defined as the width of one filament extruded by the FDM nozzle [8]. This parameter usually varies between 0.3mm and 1mm [8,18], depending on the material, FDM printer, and settings. Layer thickness is usually described as the thickness of a deposited bead, which is equivalent to the thickness of one layer [8]. Values are commonly taken as one half of the bead width. To avoid ambiguities, it can be helpful to distinguish the terms *layer thickness* and *bead height*, which are generally used interchangeably. To clarify: in this review, *bead height* refers to the height of the ellipse, while *layer thickness* refers to the actual height of one layer, that is, with the subtraction of the overlap between layers above and below, as can be seen in Figure 3.

This subtraction of the overlap between layers is usually referred to as the *overlap interval*, *hatching distance*, or *air gap*. Nevertheless, it is again debatable whether one should differentiate between these terms. The *overlap interval* is often used to define the vertical overlap between layers above and below, not between beads. The *air gap* or *hatching distance* is commonly used to describe the horizontal distance or overlap between adjacent beads. Similarly, the available literature lacks a clear definition, since all terms are used interchangeably. Most works use the aforementioned terms to describe the horizontal distance or overlap between adjacent beads. However, in [21,22,23], these terms were used for the vertical overlap between layers above and below. The most common terminology will be used here, that is, the *overlap interval* defines the vertical overlap, while the *air gap* describes the horizontal distance or overlap (see Figure 3). Regarding the horizontal distance between the beads (air gap), the standard value is mostly set to zero, such that the beads just touch [8,23] and thus do not intersect. Alternative settings are either a positive air gap, meaning that the beads do not touch and therefore leave an actual air gap, or a negative air gap, resulting in overlapping bead tracks where adjacent beads intersect (this is the case in Figure 3).

Related to the latter parameter(s) are the heterogeneities introduced by the FDM process at the micro and meso length scales, i.e., voids and pores [7], or *porosity*; see Figure 2 and Figure 3. As stated previously, these geometrical variations result in parts having the same net shape at the macroscale, while their mesostructures can be very different [24,29]. A related term is *solidity*, which is defined by the voids between neighboring beads [19] and can be used to express the so-called solidity ratio (SR). This ratio is in fact a normalization of the density, which varies depending on the bead shape and has a maximum value of 1.0, meaning a fully solid part. Porosity is therefore considered to be an important parameter that requires significant investigation with respect to the mechanical properties of FDM-produced parts [1].

Similar to the porosity, the *surface roughness* or *surface quality* is inherent to the layer-by-layer deposition of the AM process; see Figure 2b and Figure 3. The resulting surface texture can be seen as a superficial defect in FDM-manufactured parts, since the three-dimensional solid CAD model does not include this detail [21]. Because the cross-sectional shape of the beads resembles elliptic curves, variations in the surface occur [23]. In fact, these can be regarded as the ‘porosity of the edges’.

The *inclination angle* or *surface angle* is connected to the surface roughness in the sense that it influences the effective roughness, particularly when inclined surfaces are involved (see Figure 3). This parameter is known to cause and influence the stair stepping effect [23], staircasing [20], or the *staircase effect* [21] and is also a result of the layer-wise printing procedure. It is considered to be an extreme defect of the surface, which occurs when surfaces are printed under an angle (defined by this parameter) such that successive layers cannot be stacked directly on top of each other. Consequently, relatively large stair-step-shaped defects exist on inclined surfaces of 3D-printed parts, as illustrated in Figure 3.

**Figure 4:**
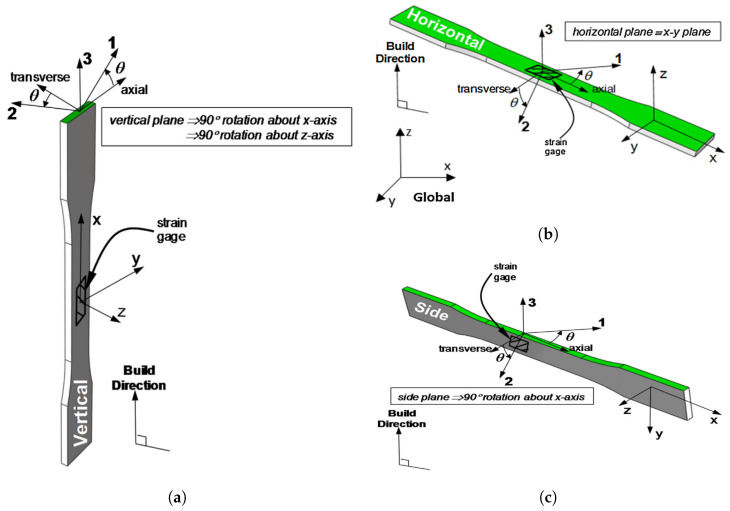
Vertical (V), horizontal (H), and side (S) build directions [13]. (**a**) Vertical build direction (V). (**b**) Horizontal build direction (H). (**c**) Side build direction (S). Reproduced with permission from Elsevier, 2016.

The *build direction* or *part orientation* is the direction along which the consecutive layers are stacked during the FDM process. By definition, this direction is orthogonal to the platform of the FDM printer (see Figure 1) and thus also orthogonal to each layer’s surface [8]. Parts built in different build directions may have deviating numbers of layers and volumetric errors (e.g., porosity or surface roughness). Three different build directions (V–H–S) of a part with respect to the Cartesian coordinate system (x−y−z) are depicted in Figure 4.

**Figure 5:**
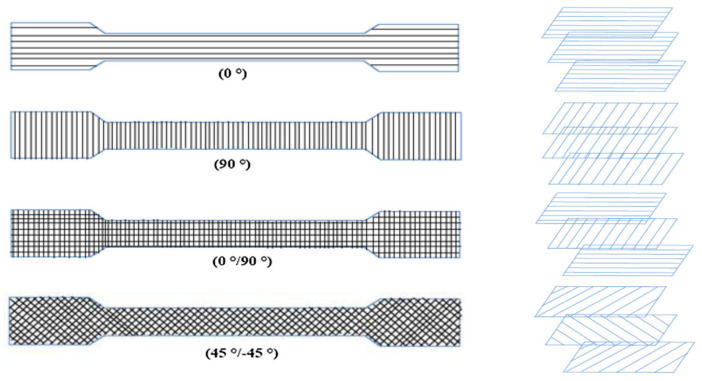
Illustration of the raster orientations for a part loaded in the horizontal direction [11]. Reproduced with permission from Elsevier, 2019.

In addition to the build direction, the *raster orientation* or *hatching angle* is known as a significant process parameter affecting the mechanical characteristics of 3D-printed parts [7]. The raster orientation is defined as the slope of the extruded filaments, usually compared to the loading direction [8,18]. Typical raster orientations are 0${}^{\circ }$, 90${}^{\circ }$, $0{}^{\circ }/90{}^{\circ }$, and $\pm 45{}^{\circ }$ (Figure 5).

Besides the internal raster discussed and depicted above, which is the infill, it is customary for an FDM 3D-printed part to have yet another component, namely the *outer shell*, composed of the *contours* and solid/bottom layers [2]. Usually, the manufacturing process starts with depositing filaments along a part’s edge [8]. After completing an entire contour, one more additional filament is often deposited inside. Depending on the defined *number of contours*, one of the printing process parameters considered here, new contours are added on the inner side of the previous contour. Afterwards, the remainder of the internal portion of the part is filled according to the raster orientation described earlier [19]. Please note that, depending on the slicing software, the outer shells of parts are usually printed in a so-called SOLID fashion [5] by default, while the internal raster may have a larger air gap to save material or reduce weight.

**Figure 6:**
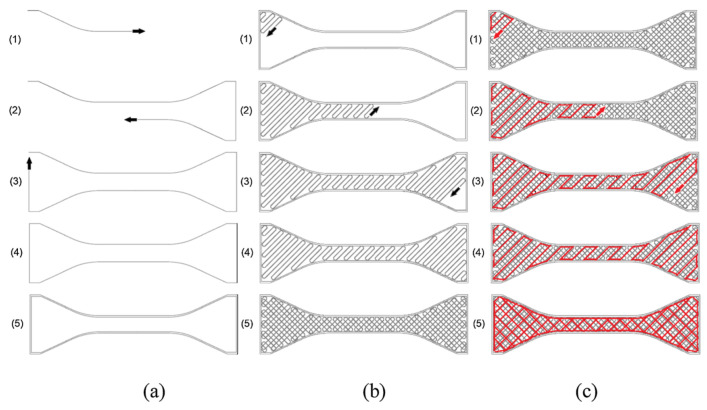
Schematic representation of printing path for the (**a**) outer shell, (**b**) bottom layer, and (**c**) infill [5]. Reproduced with permission from Elsevier, 2019.

All the consecutive motions of the nozzle for extruding the fused filament together, as part of the G-code, constitute the so-called *printing path*, the last parameter considered here. A schematic representation of the complete printing process of an FDM part is visualized in Figure 6, where the number of contours is two (Figure 6a) and the raster orientation is set as $\pm 45{}^{\circ }$ (Figure 6b). Figure 6c illustrates an infill with a larger air gap compared to the bottom layer of the part. For two-dimensional representations of 3D-printed polymers, as displayed in Figure 2, the printing path mostly influences the cross-sectional shape parameters of the beads. However, when looking at three-dimensional representations, more printing process parameters become important, like the build direction and raster orientation.

Other parameters, such as nozzle size and temperature, are recognized to have an influence on the properties of FDM-printed parts. Nevertheless, parameters other than those listed in this section are either difficult to control or assumed to be related to those already considered. For example, nozzle size mainly affects the cross-sectional parameters of the beads, while temperature (e.g., thermal history) is very difficult to control as it is a function of both location and time [35]. Several publications have aimed to either optimize the printing process parameters or apply external modifications to the 3D-printing process. According to [39], these can be subdivided into (i) pre-process, (ii) in-process, and (iii) post-process methods. An example of this first strategy is to modify the material prior to printing by adding a low-molecular-weight (LMW) additive [41]. In-process technique examples can be found in the form of nozzle modifications, like adding hot air nozzles [43] or a pre-heater [44], or using a laser system [45] to locally heat the previously deposited layer. Post-process methods often include treatments of various kinds, such as acetone vapor [6].

## 3. Characteristic Mechanical Properties

As described in the previous sections, 3D-printed polymers contain heterogeneities at the micro and meso length scales, causing anisotropy in the produced parts [3,6,9,13,14,18,19,29,37]. The resulting mechanical properties are often uncertain [8] and can be greatly influenced by small changes in any of the previously discussed printing process parameters. Most studies have shown that the mechanical properties are negatively affected. In this section, the characteristic mechanical properties of 3D FDM-manufactured parts are listed, including (some of) their most influential parameters.

The most studied mechanical property is *strength*, specifically *tensile strength*, since it is most influenced by the printing process parameters. According to Ahn et al. [18], the raster orientation and air gap have a large effect on the tensile strength, while the bead width was found to have only a small influence. A recent study using machine learning (ML) models to predict relationships between printing process parameters and mechanical properties [46] found that larger infill densities (related to the air gap) have a positive effect on tensile strength, while higher printing speeds negatively affect this mechanical property. Furthermore, *compressive strength* was found to be unaffected. Generally, studies on compressive strength are very rare, and the only examples we found were [18], as mentioned above, and [4], in which the tension/compression asymmetry and direction-dependent response of the material were discussed.

**Figure 7:**
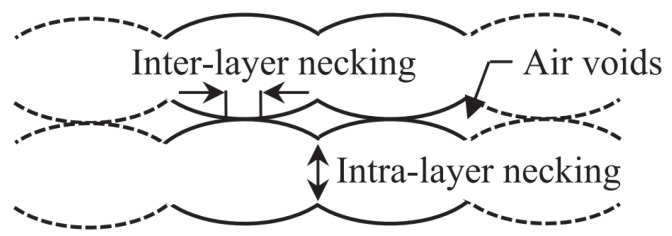
Inter-layer and intra-layer necking [6]. Reproduced with permission from Elsevier, 2017.

Related to strength are two other characteristic mechanical properties found in literature by which FDM parts are deeply affected: the *inter-layer* and *intra-layer bonding* between the beads [9,25]; see Figure 7. Together with the heterogeneities resulting from FDM (i.e., air voids), weak inter-layer adhesion is one of the most significant problems of this process [42]. The results in [30] indicate that the average strength of intra-layer bonds is higher compared to that of inter-layer bonds. The failure of FDM-manufactured parts was found to mainly occur along layer interfaces [6], mostly related to the level of adhesion between different layers, and it is clear that raster orientation has a great influence. Additionally, this level of adhesion was found to be dependent on various printing process parameters, with temperature (i.e., thermal history), which is very difficult to control, being the most influential [35]. As defined by Yao et al. [15], inter-layer failure occurs at the interface of two neighboring layers, meaning that the material layers remain undamaged afterwards. To approximate this interface strength, the tensile strength of vertically built parts is sometimes used [28]. However, in general, the adhesive strength between layers cannot be obtained directly from stress–strain-based methods [32,33], nor do these methods have predictive capabilities [32]. Inter-layer failure is known to negatively affect the *tensile failure strength* (TFS) [15], which is defined as the strength perpendicular to the boundary between two layers. A very recent study by Monaldo and Marfia [25] explained the inter-layer bond as *cohesion* between neighboring layers (i.e., vertically) and the intra-layer bond as *cohesion* between neighboring beads inside the same layer (i.e., horizontally), rather than using the more common description involving *adhesion*. They attributed the early failure of 3D-printed parts to de-cohesion between deposited beads, where the inter-facial bond strengths are governed by the printing process parameters.

Besides strength, *stiffness* is also significantly affected by the printing process parameters. For instance, Croccolo et al. [8] observed that a larger number of contours yielded higher stiffness. Furthermore, the results obtained in [11] illustrate that it is inadequate to use the volume fraction of pores to extrapolate the stiffness of printed parts from the stiffness of injection-molded samples and the porosity volume fraction.

Another typical property that characterizes the mechanical response of FDM-printed parts is the *modulus of toughness*, which was found to increase under higher infill densities [5] and could be related to a greater elongation at break [13]. Additionally, build direction and raster orientation were found to have a significant impact [29]. Toughness is usually measured as the area underneath the stress–strain curve [38].

Other mechanical properties include the *Poisson ratio*, which is often found to be approximately equal to 1/3 [7]; the *shear strength* or *shear failure strength* (SFS) [15], defined as the strength at the boundary between two layers of material; and the *deflection at failure*, which was found to be correlated with infill density and directional parameters [28]. Table 1 summarizes the characteristic mechanical properties described in this section with the influencing printing process parameters, including literature references.

## 4. Available Characterization Techniques and Models

An extensive literature review on modeling strategies for FDM-printed parts was performed by Paul [1]. In this work, the author concluded that existing (finite element) models for FDM research are far from realistic and not computationally efficient. According to Paul [1], available (numerical) models can be partitioned as follows: (i) thermal analysis, (ii) geometrical analysis, and (iii) mechanical characterization. An extensive tabular summary can be found in the aforementioned reference. Nonetheless, Sharafi et al. [28] categorized the available literature as: (i) exploratory experimental studies on the relation between the printing process parameters and mechanical characteristics of 3D prints, (ii) numerical investigations and simulations that consider the macroscopic behavior in relation to a restricted number of printing process parameters, and (iii) extensive finite element analyses of mechanical behavior without considering the direct effects of printing. Finally, the review presented here considers geometrical analyses, numerical analyses, and experimental analyses. The latter is included for completeness and because most (numerical) studies are accompanied by experimental validations. Thermal analyses are beyond the scope of this review. Table 2, at the end of this section, summarizes for each characteristic mechanical property which printing process parameter negatively affects the microstructural composition of 3D-printed polymers.

### 4.1. Experimental Analysis

**Figure 8:**
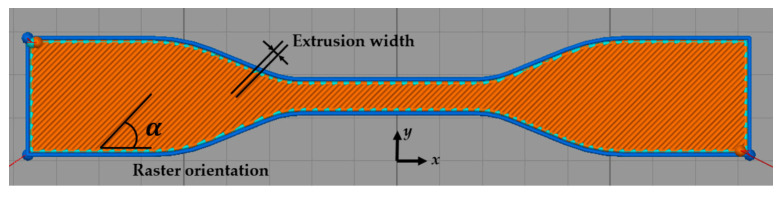
Tensile test specimen including printing path, raster orientation, and extrusion width [7]. Reproduced with permission from MDPI, 2019.

Almost all experimental analyses performed on 3D-printed FDM parts are tensile tests, e.g., [2,3,4,5,6,7,8,11,13,15,18,19,27,28,34,38]. These experiments are conducted on the specimen scale, specifically with ASTM dog bones in accordance with the testing guidelines ASTM D638 [47]. Usually, the raster orientation is considered to be a major influential process parameter. An illustration of such a tensile test specimen, including a printing path with indications of raster orientation ($\pm 45{}^{\circ }$) and extrusion width (bead width), is shown in Figure 8. Other common test methods for 3D-printed polymer structures include (three-point) bending (and impact) specimens [34].

**Figure 9:**
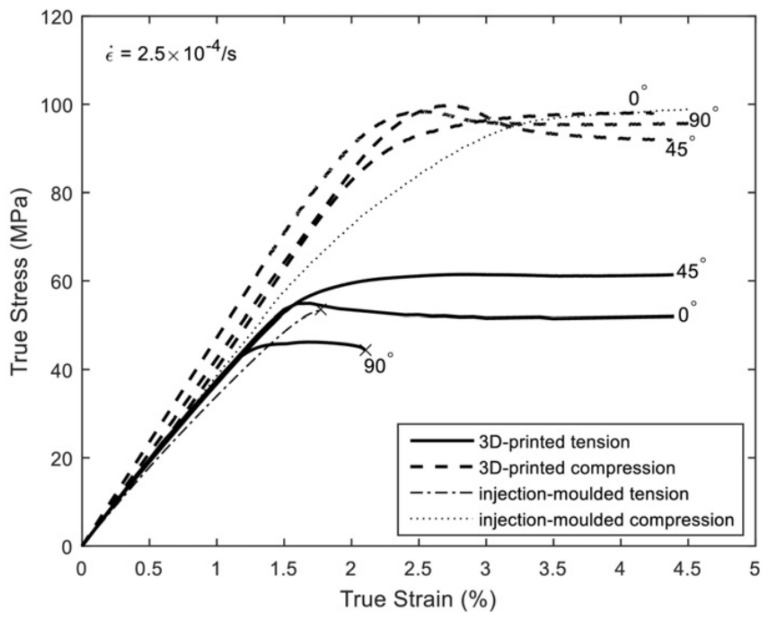
Tensile and compressive response of test specimens for various build directions [4]. Reproduced with permission from Elsevier, 2017.

Other experiments are often performed alongside tensile tests. In the literature reviewed in this study, only two reports of compressive tests performed alongside tensile tests were found, i.e., [4,18]. In the compressive experiments performed in [18], cylindrical specimens were tested, only examining the build direction. The results showed higher compressive strength compared to the tensile strength obtained in the same work. In the other study [4], compression tests were conducted by loading cuboidal specimens. Similarly, the response of the material exhibited tension/compression asymmetry and direction-dependency. The authors concluded that the elastic response (i.e., stiffness) was approximately the same in terms of tension and compression, regardless of the test direction. All tests showed ductile elasto-plastic behavior in terms of both tension and compression; see Figure 9.

**Figure 10:**
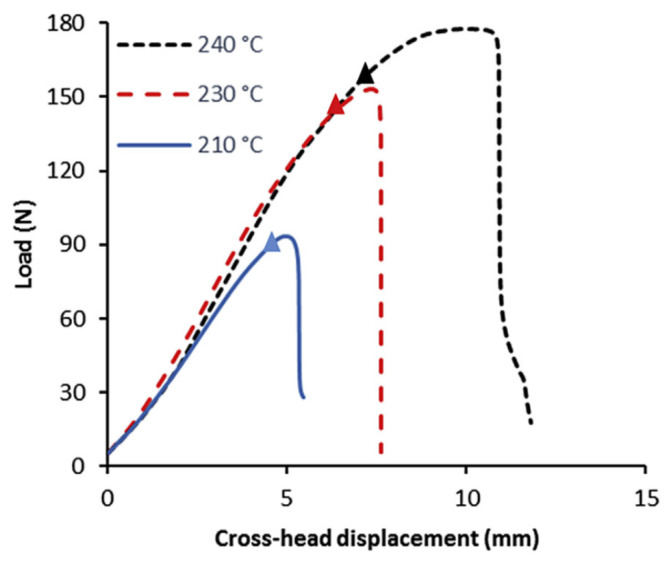
Load versus cross–head displacement results for a variety of extrusion head temperatures (cracks started to occur at the triangular indicators) [32]. Reproduced with permission from Elsevier, 2017.

Different experimental studies have analyzed fracture toughness for different mesostructures, e.g., three build directions and two raster orientations [29], or between two materials, e.g., with different degrees of stiffness [40]. Compact tension specimens, according to ASTM D5045 [48], are used to realize Mode I crack opening for the determination of fracture toughness. Representative load versus crack opening curves have been obtained, from which it could be concluded that build direction and raster orientation have a great influence on fracture toughness. Similar experiments were reported in [32,33] using double-cantilever beam (DCB) fracture specimens whose raster orientation was in the same direction as the crack growth to enable the measurement of the (adhesive) bond strength between layers. To achieve this, the real load-bearing cross-section was measured by optical microscopy, referred to as the surface intact ratio, which is the ratio between this real cross-sectional portion and the nominal fracture surface area (based on the bulk geometrical dimensions). Representative load–displacement curves have been obtained for various printing temperatures; see Figure 10.

**Figure 11:**
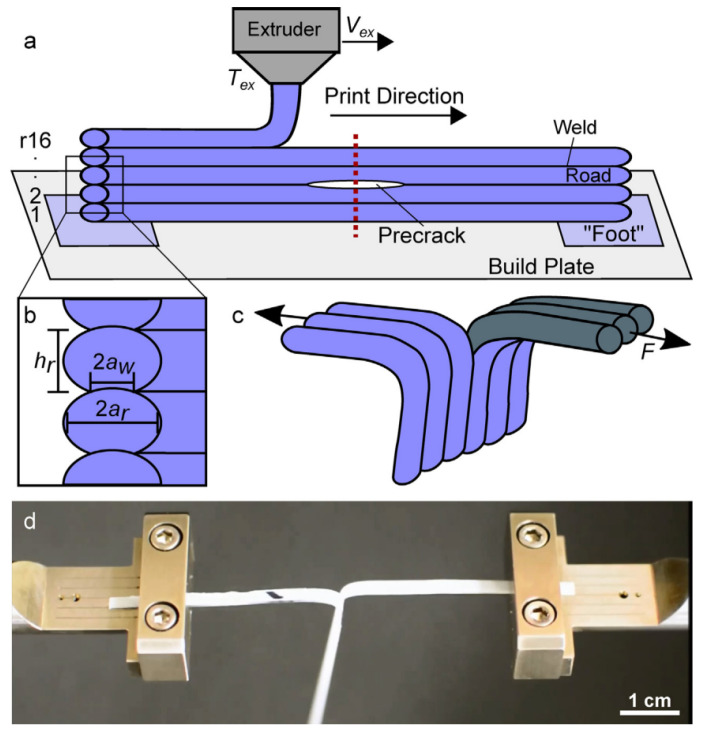
Schematic of sample preparation and experimental setup [31]. (**a**) Specimens of one single bead in thickness, including layer indications (r1–r16) and dashed cutting line. (**b**) Close up of specimen section with symbolic indications for bead height, $h_{r}$, bead width, $2a_{r}$, and weld width, $2a_{w}$. (**c**) Test setup. (**d**) Picture taken during experiment. Reproduced with permission from Elsevier, 2017.

One particular Mode III ‘trouser tear’ fracture experiment, modified from the ASTM D1938-14 [49] test method, was used to determine the strength and fracture toughness of a single bond (referred to as a ‘weld’) [31]. Similar to the DCB specimen studies above [32,33], this experimental study also used optical microscopy for cross-sectional characterization, i.e., obtaining the actual weld dimensions. Figure 11 shows a schematic of the sample preparation process as well as the experimental testing setup. Representative force versus cross-head displacement curves were obtained for the different printing temperatures and printing velocities and were used to determine an average tearing force. Furthermore, another self-developed test setup for the determination of the inter-layer shear bond strength was reported in [39], in which a sharp tip was used to tear the top layer from a substrate while the tearing force, representing the inter-layer bond strength, was measured.

### 4.2. Finite Element Analysis

The influences of numerous printing process parameters on the characteristic properties of FDM parts, such as those discussed in the previous section, govern the finite element analysis (FEA) in this area [1]. In this portion of the literature review, special attention is paid to FEA that specifically considers the microstructural effects resulting from the process parameters to model the material as a continuum, either through the use of a representative volume element (RVE) or by modeling the specimen completely.

However, it should be noted that until recently no FEA studies *explicitly* modeled the inter-layer or intra-layer bonds. Rather, they considered the geometry resulting from the FDM process, meaning that the main focus was on modeling the characteristic cross-sectional shape (see Figure 2), including its related heterogeneities, i.e., voids and pores. Thus, the aim was to indirectly incorporate the effects caused by the different layer bonds. To this end, either porosity was included in the geometrical representation of the material to obtain the so-called ‘bonded regions’ [6], or the bonded dimensions between the filaments in the RVE were modified according to the experimental results [28], but inter-layer and intra-layer bond properties (e.g., strength) were never considered simultaneously. Instead, the material was generally considered to have the same properties in these ‘bonded regions’. Only some recently published FEA studies using phase-field models for 3D-printed materials actually distinguished between cross-layer or bulk layer failure (i.e., filament or bead failure) and inter-layer or inter-phase failure [50,51] in their formulations. Similar observations were made in a very recent publication by Monaldo and Marfia [25], who, to the best of the authors’ knowledge, were the first to *explicitly* incorporate both the porosity and inter-facial bond properties (i.e., inter-layer and intra-layer bond strengths) simultaneously in numerical (microstructural) models for 3D-printed polymers produced by material extrusion.

#### 4.2.1. Representative Volume Elements

Three different approaches are distinguished in the literature when it comes to FEA using RVEs based on (i) quantitative characterization by X-ray computed tomography (XCT); (ii) microstructural images and cross-sectional morphology (e.g., actual versus idealized RVE); and (iii) homogenization approaches (e.g., Mori–Tanaka).

##### Quantitative Characterization by X-ray Computed Tomography

**Figure 12:**
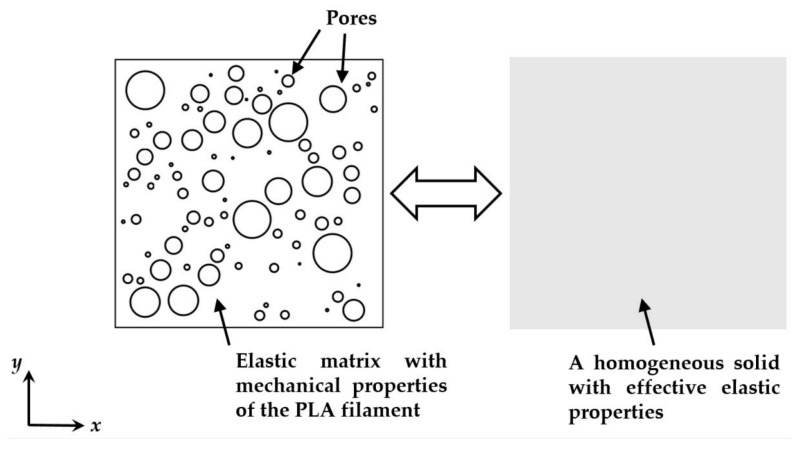
Representative volume element and equivalent homogeneous solid [7]. Reproduced with permission from MDPI, 2019.

In this case, a micromechanical FEA was used for the prediction of elastic properties based on the microscopic details of an actual 3D-printed specimen that was characterized by XCT [7]. This model estimated the macroscopic response of the material via the two-dimensional analysis of a periodic RVE. The internal structure was envisioned as a square elastic matrix, where the pore size and distribution were determined from the actual pore sizes obtained by XCT, and the pores were assumed to be circular in shape; see Figure 12. In the end, predicted Young’s modulus versus porosity curves were obtained that were generally close to those of other models.

##### Microstructural Images and Cross-Sectional Morphology

**Figure 13:**
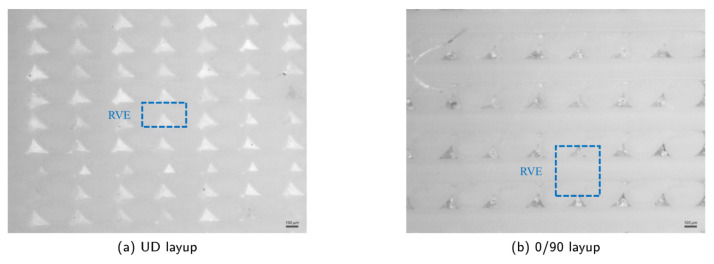
Microstructural images of different layups including actual RVEs [14]. Reproduced with permission from Elsevier, 2020.

Most FEA studies belong to this category, in which microscopic images are usually employed to acquire RVEs. Images obtained in [14] clearly showed inter-bead voids that were inherent to the printing process; see Figure 13. Two separate RVEs were selected for two different raster orientations, namely (a) a unidirectional (UD) layup, and (b) a 0/90 layup. In addition to the actual RVEs shown here, ideal RVEs were also directly obtained from the slicer software to study the influence of the microstructure. It was found that the use of actual RVEs had no advantages compared to using ideal RVEs for the 0/90 case. Similar to the model using quantitative characterization by XCT (i.e., [7]), Young’s modulus versus air gap (related to porosity) curves were constructed using the models in [14]. However, only two raster orientations were investigated in [14], while [7] considered four variations in raster orientation.

**Figure 14:**
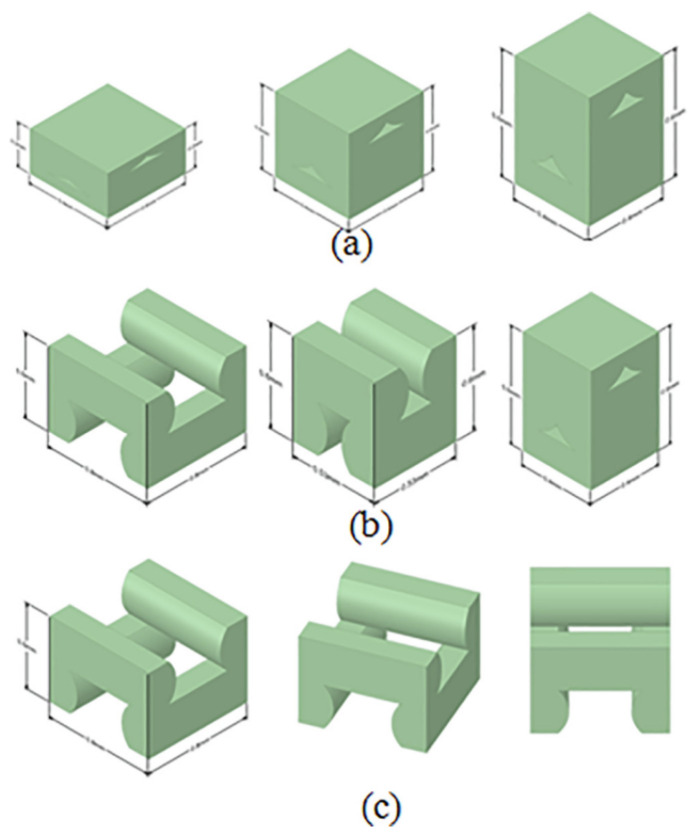
Idealized RVEs for (**a**) negative air gap and different layer heights, (**b**) constant layer height and different air gap values, and (**c**) positive air gap and different raster orientations [9]. Reproduced with permission from Elsevier, 2020.

Other studies started directly from an idealized RVE, in which the cross-sectional morphological characteristics of FDM parts (such as voids) were observed to occur in a repetitive pattern [9]. In [9], the effect of a variety of printing parameters on the mechanical properties were investigated by altering the layer thickness, raster orientation, and air gap. The RVE was modeled assuming that the inter-layer boundaries were completely fused with adjacent material, which is arguably an overestimation of the inter-layer bond strength. The considered ideal RVEs are depicted in Figure 14. Again, the results suggested a relationship between the elastic modulus and air gap (i.e., porosity). Additionally, relationships for both the elastic and shear moduli were obtained with respect to all the considered printing parameters.

##### Homogenization Approaches

**Figure 15:**
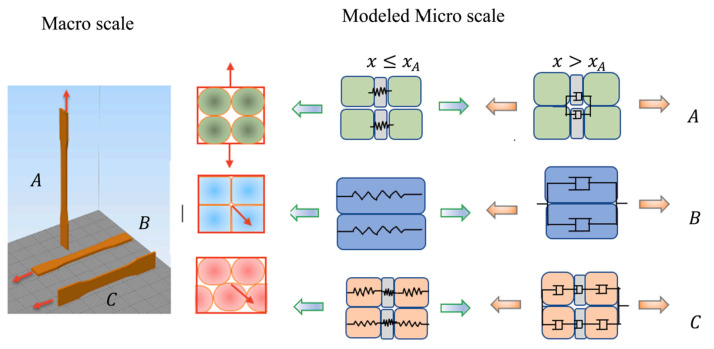
Schematic representation of the load transfer between the macro- and microscale for three different build directions [28]. Reproduced with permission from Elsevier, 2022.

For this purpose, a phenomenological numerical model is used to compute local stiffness matrices from an RVE [28]. In this specific study, raster orientation, build direction, and inter-facial bond dimensions (which are related to layer thickness, bead width, overlap interval, and air gap) were taken into account at the microscale. To characterize the material at the macroscale by making use of the Mori–Tanaka homogenization framework, the model considered the RVE as springs and dashpots to include both elastic and plastic responses. The effect of porosity (i.e., voids) was included by reducing the RVE’s stiffness for larger porosities. Figure 15 presents a schematic representation of the load transfer between the macro- and microscale for three different build directions. The results of the experiments were used to model the geometry of the RVE. As shown in Figure 15, the material response was elastic until yielding occurred, i.e., $x\leq x_{A}$, after which plastic deformation occurred, i.e., $x>x_{A}$.

**Figure 16:**
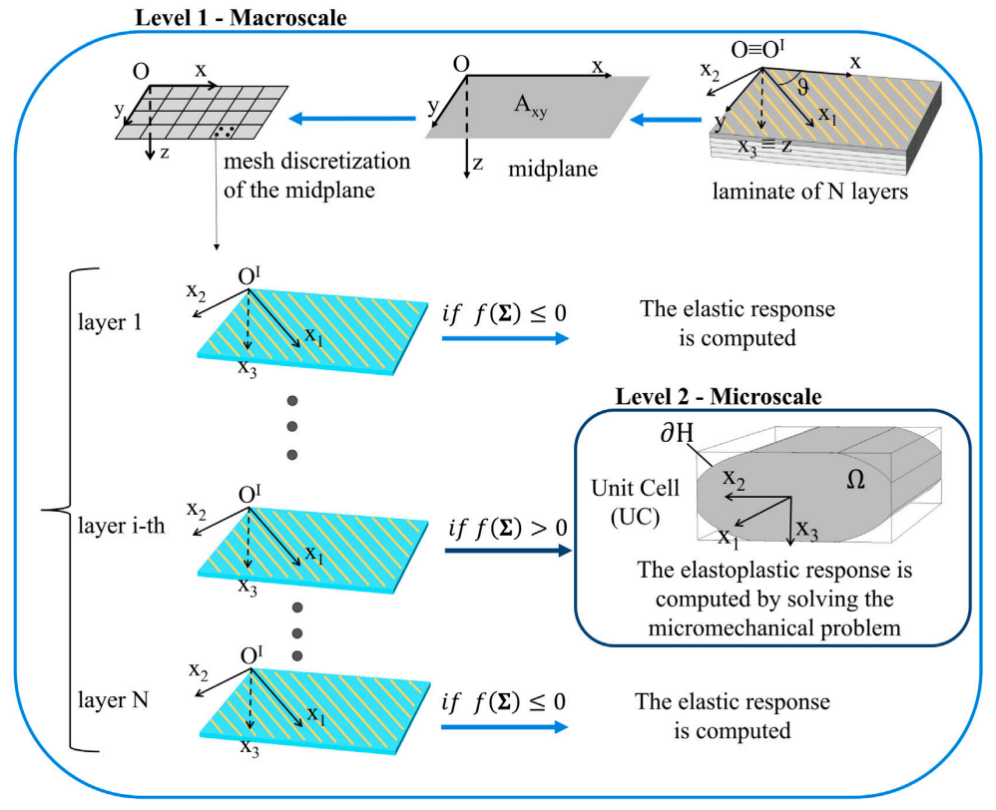
Schematic description of the two-level strategy for multi-scale analysis [27]. Reproduced with permission from Elsevier, 2023.

More recent developments by Monaldo et al. [24,25,27] included promising multi-scale approaches that considered the cross-sectional shape of the microstructure using a reduced-order model (ROM) while performing macroscopic homogenization by means of first-order shear deformation laminate theory (FSDT). To represent the microstructure of 3D-printed polymers, these models considered pseudo-elliptical bead shapes including porosity (referred to as unit cell (UC)); see Figure 16. Initially, the authors used geometrical data from Alaimo et al. [2] and Garg and Bhattacharya [6] in [24], while microscopic images were used in [27]. Previous modeling attempts assumed a constant layer height and perfect inter-layer bonding, although changes in raster orientation were permitted in each layer [24]. Further assumptions in these two-level multi-scale models included that the bead phase (i.e., material parallel to the printing path) behaved elasto-plastically and exhibited locally homogeneous and isotropic behavior [27]. A major drawback in the earlier multi-scale models proposed by Monaldo et al. [24,27] was that they did not consider damage phenomena like inter-layer and intra-layer bond failure, which were identified from the plastic deformation results in [27].

**Figure 17:**
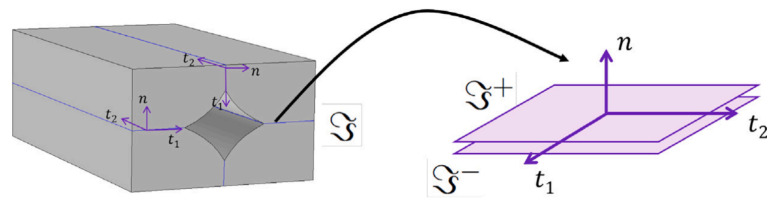
Schematic representation of the interface and its local coordinate system [25]. Reproduced with permission from Elsevier, 2023.

Nevertheless, in their latest publication, Monaldo and Marfia [25] realized the importance of capturing the inter-layer and intra-layer (de-)cohesion when simulating the strength of 3D-printed polymers produced by material extrusion. To this end, they extended their former multi-scale models by introducing a cohesive damage interface model using a cohesive constitutive relationship through transformation field analysis (TFA). In this case, the UC (or RVE) was described by a parallelepiped including four quarters of the previous pseudo-elliptical bead shapes with a void at its center (see Figure 17). Similar to other works, the bead phase was described by elasto-plastic behavior (e.g., [2,24]). The cohesive damage interface model enabled the inclusion of de-bonding and cohesive cracking among neighboring beads by means of zero-thickness interfaces. Monaldo and Marfia [25] also addressed the limited availability of inter-facial properties, which is duly noted by the authors. Therefore, they assigned inter-facial stiffness and strength properties based on assumptions. The results indicated intra-layer de-cohesion, with most damage occurring near the void. Contrary to most reports in the literature, inter-layer damage did not appear. Furthermore, it is worth mentioning that all models proposed by Monaldo et al. [24,25,27] achieved significant savings in computational demand (i.e., internal or historic variables) compared to non-linear FEA (e.g., FE^2^). To the best of the authors’ knowledge, the multi-scale technique proposed by Monaldo and Marfia in [25] was the first to *explicitly* incorporate both the porosity and inter-facial bond properties (i.e., inter-layer and intra-layer bond strengths) simultaneously in a numerical (microstructural) model for 3D-printed polymers produced by material extrusion.

#### 4.2.2. Specimen-Level Models

**Figure 18:**
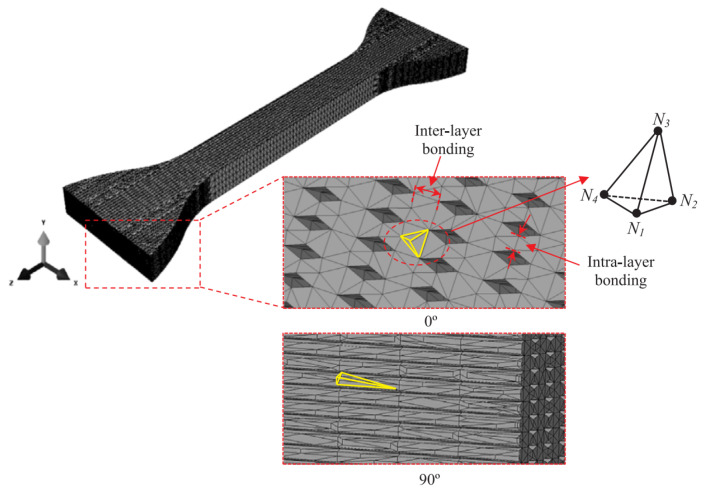
Three-dimensional tensile test specimen model, including mesh and elements [6]. Reproduced with permission from Elsevier, 2017.

Several studies have focused on modeling a complete 3D-printed specimen with FEA [5,6,28]. However, only Garg and Bhattacharya [6] have reported a realistic geometrical representation of an FDM tensile test specimen (ASTM D638), which was created in accordance with microscopic images. The entire specimen was modeled in 3D, only excluding the regions under the grip in the tensile testing machine to reduce computational costs. As a result, various printing process parameters had to be included in the model, such as raster orientation, layer thickness, overlap interval, and air gap. Isotropic and homogeneous material behavior was assumed, based on experimental data for solid samples. The entire three-dimensional tensile specimen model can be seen in Figure 18. It was observed that necking occurred for raster orientations parallel to the loading direction, and inferior strength was obtained for specimens with a build direction parallel to the direction of loading.

**Figure 19:**
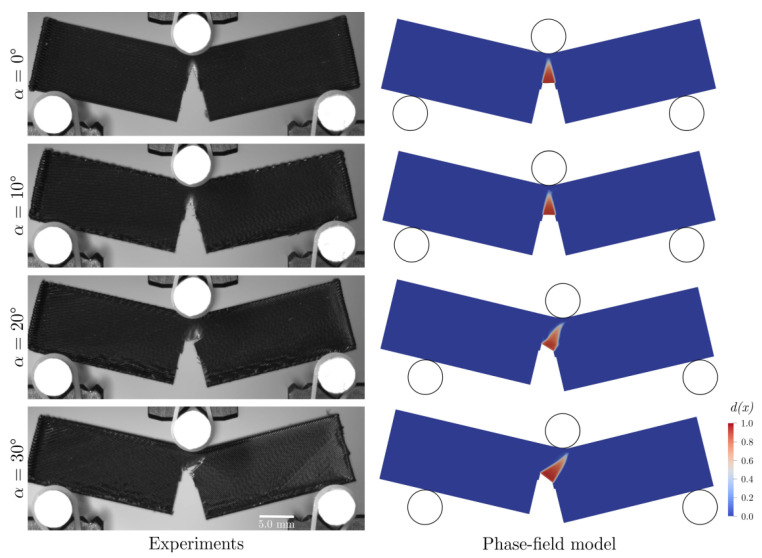
Experimental and phase-field model crack paths from 3-point bending specimens [50]. Reproduced with permission from Elsevier, 2023.

Some recently published FEA studies using phase-field models for 3D-printed parts also considered complete specimens [50,51,52,53]. The printing process parameter investigated in these studies was raster orientation. Most of these studies were limited to two-dimensional representations, but Khosravani et al. simulated both 2D [52] and 3D specimens [53]. Li et al. [51] presented an elasto-plastic phase-field model that incorporated anisotropy through damage variables for the bulk material (i.e., filament or bead failure) and inter-layer direction, while Khosravahni et al. [52,53] utilized anisotropic cohesive phase-field models. The latest publication by Lampron et al. [50] proposed a combination of the former two phase-field models, resulting in an anisotropic fracture model comprising cross-layer fracture (i.e., filament or bead failure) and inter-layer fracture (similar to Li et al. [51]) without an explicit consideration of interfaces. The quantitative and qualitative experimental findings from three-point bending specimens agreed well with the numerical results for different raster orientations; see Figure 19.

### 4.3. Analytical and Theoretical Models

Two types of analytical and theoretical models are distinguished for 3D-printed materials in this study, namely yield criteria and plasticity models and predictive and generalized strength models. In what follows, some notable models found in the literature will be discussed.

#### 4.3.1. Yield Criteria and Plasticity Models

Three-dimensionally printed materials have already been modeled using classical lamination theory (CLT) and the Tsai–Hill yield criterion in several studies [2,15], where layers of parallel beads are considered as lamina. The material strength of AM parts is incorporated through the Tsai–Hill yield criterion for the multi-axial loading of composites. The resulting expression can be used to compute the material’s yield strength with respect to raster orientation. Furthermore, an equation for obtaining the elastic modulus with respect to raster orientation for unidirectional printed parts is derived, as well as insight into strain at failure, shear modulus, and shear strength [2]. Another strategy for modeling the plastic and damage behavior of 3D-printed parts was presented by Kerekes et al. [5], using a Gurson–Tvergaard (GT) yield function for porous plastic materials and inverse analysis. This yield function decreased with increasing pore volume fractions, enabling the inclusion of softening behavior.

#### 4.3.2. Predictive and Generalized Strength Models

**Figure 20:**
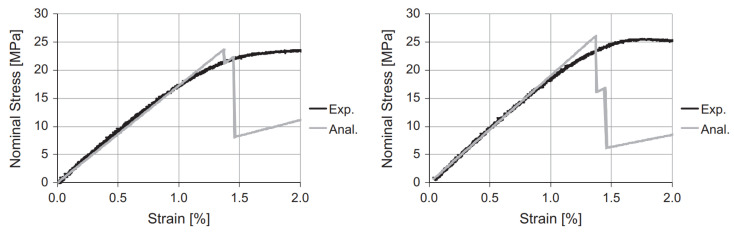
Predictive analytical model compared to numerical and experimental results [8]. Reproduced with permission from Elsevier, 2013.

Croccolo et al. [8] addressed the availability of *predictive models* that account for important printing process parameters and are capable of predicting the mechanical properties of 3D-printed parts, specifically referring to strength and stiffness. To this end, an analytical model was developed to match experimental findings by incorporating influential parameters like part dimensions, build direction, bead width, raster orientation, layer thickness, and the number of contours. These types of models can be particularly useful in the design of FDM-printed components to avoid the extensive tuning of printing parameters like the air gap and raster orientation, which are usually set to default values in the production process. The model considered here could provide strength and stiffness characteristics given the aforementioned parameters. This is shown in Figure 20, where it can be seen that the predictive analytical model corresponded particularly well in the initial stage to the experimental results.

Another approach is represented by generalized strength models for predicting the tensile failure strength resulting from inter-layer failure in 3D-printed parts [15]. Previously, it was concluded that the failure of FDM-manufactured parts mainly occurred along layer interfaces (see Section 3). Therefore, the strength model developed in [15] was based on this characteristic of 3D-printed polymers and included TFS (as defined in Section 3) as well as *shear failure strength* (SFS). TFS and SFS can be determined from experiments to obtain expressions for failure, equivalent to an inter-layer failure-informed failure criterion. Similar to the yield criteria and plasticity models discussed above, a generalized strength model can predict the inter-layer failure of FFF 3D-printed materials as a function of raster orientation.

### 4.4. Microstructural and Fractographic Analyses

**Figure 21:**
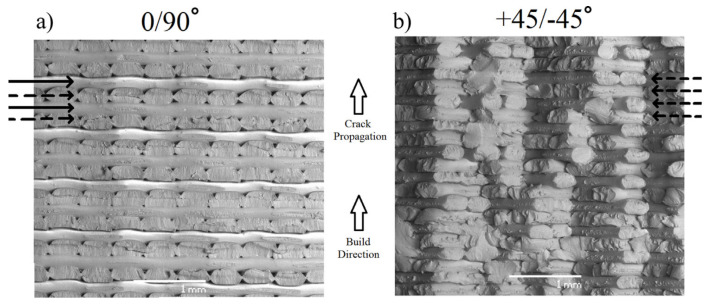
SEM fracture surfaces for two different raster orientations: (**a**) $0^{\circ }/90^{\circ }$ and (**b**) $\pm 45^{\circ }$ [29]. Reproduced with permission from Elsevier, 2017.

In order to gain more insight into the printing process parameters affecting the failure mechanisms of 3D prints and the geometric characteristics of their microstructures, a variety of microstructural and fractographic analysis techniques are available. A vast majority of this information is acquired through scanning electron microscope (SEM) pictures, e.g., [4,6,11,18,29,38], of which an example has already been shown in Figure 2 from [18]. For instance, by considering the fracture surfaces obtained in [29] (see Figure 21) it was concluded that $0{}^{\circ }/90{}^{\circ }$ specimens failed in a brittle manner, presenting a planar fracture surface (Figure 21a), while the $\pm 45{}^{\circ }$ specimens showed a more jagged saw-toothed topography, resulting in more energy dissipation, which increased the fracture toughness (Figure 21b). In Figure 21, dashed arrows indicate shear failure and solid arrows illustrate inter-filament failure.

Other types of resources include microphotographs [12] or optical microscopy images [14], photographic images [13], and XCT [7,10]. A microphotograph taken from [12] was shown earlier in Figure 2a. These resources can be used for fractographic analysis, but their primary use is in determining the effect of process parameters like cross-sectional shape parameters.

### 4.5. Geometrical Analysis

Geometrical analysis is considered to be the most important characterization and modeling strategy for representing and idealizing the microstructural geometries of 3D-printed polymers. However, Paul [1] concluded in his extensive literature review that surface quality and geometry defects in FDM-printed parts are the least researched aspects in available publications and that only a few defects have been identified. Furthermore, no finite element models exist that consider defects in the geometry and surface finish of FDM-manufactured samples. According to Paul [1], various geometrical models have been suggested in the existing literature concerning the evaluation of edge quality and surface quality. This section reviews two specific types of geometrical analysis found in the literature, namely edge profile simulations and surface roughness and quality models.

#### 4.5.1. Edge Profile Simulations

**Figure 22:**
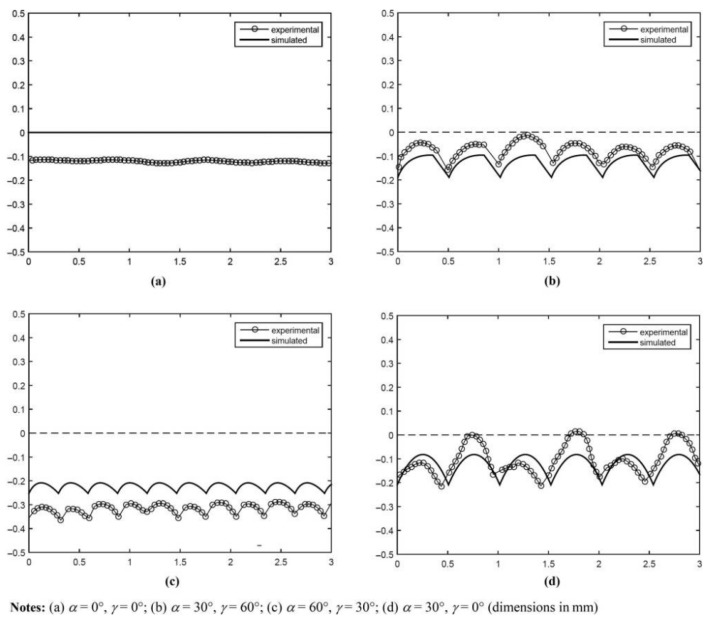
Comparison of measured and simulated profiles for constant layer thickness and β [20]. Reproduced with permission from Emerald Publishing Limited, 2019.

Edge profiles can be described as surface errors along the edges of 3D-printed parts resulting from the printing technique. These usually comprise geometric effects that cause differences compared to the intended CAD model, such as the staircase effect discussed above. Based on earlier work, Armilotta [20] presented a graphic and numerical approach for edge profiles in FDM prints in order to simulate position and form errors [1]. In this method, the edge profile was modeled based on layer thickness and three characteristic angles (inclination angle α, included angle β, and incidence angle γ). To illustrate the exactness of the simulation with respect to the shape and size of the edge profiles, Figure 22 compares experimentally measured profiles with simulated profiles for several different combinations of characteristic angles. Figure 22a depicts a horizontal edge, Figure 22b shows a stair-stepped edge with partial drag, Figure 22c displays a stair-stepped edge with complete drag, and a side-facing edge can be seen in Figure 22d. It was concluded that the shape of all edge profiles was simulated with sufficient correctness, except for the side-facing edge in Figure 22d. The term *drag* refers to the so-called drag effect that is assumed to be caused by the extruded bead sticking more strongly to the underlying deposited layer than to the metal printer nozzle. Stair stepping has already been discussed in Section 2.

#### 4.5.2. Surface Roughness and Quality Models

**Figure 23:**
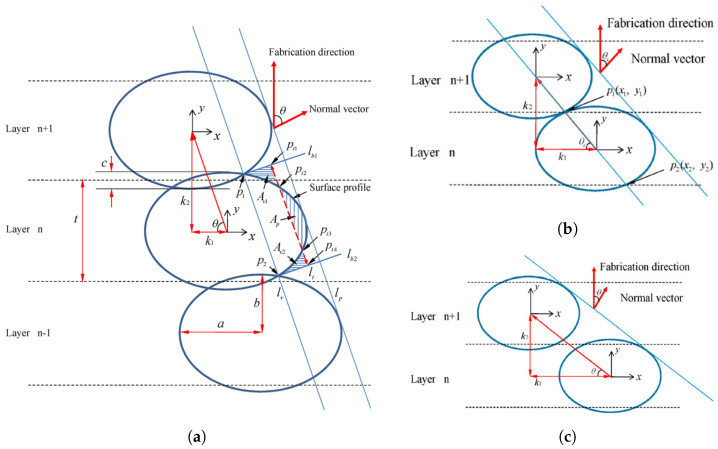
Representation of the geometric model by Ahn et al. [23]. (**a**) Representation of geometric model. (**b**) In the case of a single point of contact ($\theta_{c}$). (**c**) In the case of no intersection or contact. Reproduced with permission from Elsevier, 2009.

According to [21], the elliptic cross-sectional shape of deposited beads suggested by Ahn et al. [23] is the most realistic approximation. This model approached the surface profile shape as a series of elliptic curves that overlap each other when stacked in the vertical direction, representing the deposited layers [21], as observed in Figure 2 in Section 2. First, an expression for the surface profile was defined through the schematic representation in Figure 23a. From this figure, it can be observed that the surface profile depends on a variety of printing process parameters, namely the surface angle (θ), cross-sectional shape (*a*, *b*), layer thickness (*t*), overlap interval (*c*), and bead width (2a). Eventually, these process parameters were used to model the surface roughness. However, at some point the elliptic curves only have a single point of contact for a specific surface angle [23]; see Figure 23b. This surface angle value is defined as the critical angle ($\theta_{c}$) and relies on the previously listed parameters. If this angle is below the critical angle value, neighboring beads do not cross or touch, as illustrated in Figure 23c, and the surface roughness cannot be established. Ahn et al. [23] referred to this region, where the surface angle is smaller than $\theta_{c}$, as the ‘indeterminate region’. In the end, the simulated surface roughness values were compared with experimental data, which confirmed the validity of the obtained expression, proving its remarkably realistic approximation of the cross-sectional shape of FDM-printed parts. Haque et al. [22] used this model to show that the surface roughness is mostly affected in a negative way by the layer thickness, while other printing parameters were found to have a weaker effect.

**Figure 24:**
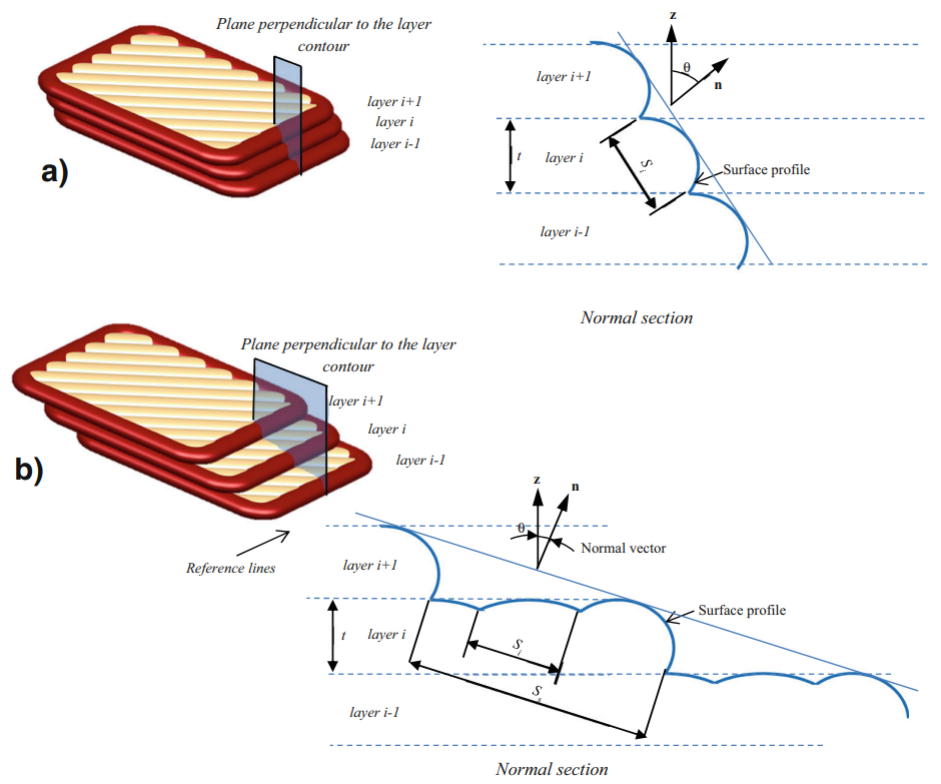
Surface profile contours for (**a**) $\theta >\theta_{s}$ and (**b**) $\theta <\theta_{s}$ [21]. Reproduced with permission from Springer Nature, 2017.

However, as mentioned above, this geometric model is not defined if adjacent ellipses do not intersect [21] (see Figure 23c). In other words, the model works for surface angles $\theta \geq \theta_{s}$. Based on the same concept as the model of Ahn et al. [23] using elliptic curves to approximate the surface profile, an extension of this model was proposed in [21] that was also defined for surface angles smaller than the critical surface angle value, i.e., $\theta <\theta_{s}$. Similarly, the evaluation of the surface profile was performed in a normal section that was orthogonal to the contours. Figure 24 illustrates this for (a) $\theta >\theta_{s}$, which is similar to the model in Figure 22, and (b) $\theta <\theta_{s}$, where the stair-stepping effect comes into play.

**Figure 25:**
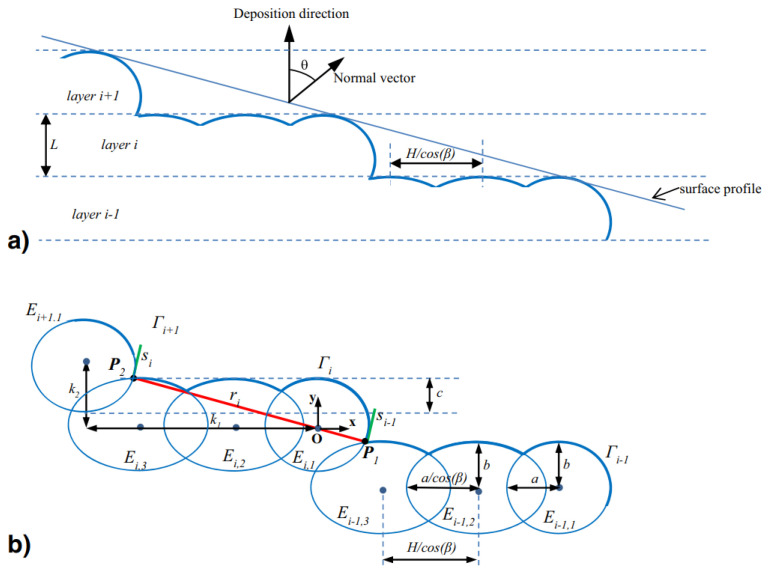
Representation of the extended geometric model by Angelo et al. [21]. (**a**) Approximation of surface profile with ellipses. (**b**) Theoretical model definition of surface profile. Reproduced with permission from Springer Nature, 2017.

For $\theta <\theta_{s}$, the surface profile is defined by steps that are composed of several ellipses, depending on the surface angle (θ), layer thickness (*L*), overlap interval (*c*), air gap (*H*), raster orientation (β), and cross-sectional shape parameters (*a*, *b*) or the ratio between the bead’s long and short axes (r=a/b). The resulting expression of the surface profile is defined by the schematic model shown in Figure 25. However, it should be noted that the model works under the assumption that a negative air gap is used, meaning that the hatching part of the layers intersects with adjacent beads. This is not always the case in practice, but it can be defined in the printing process parameters. Concerning the input parameters, layer thickness, air gap, and raster orientation can be directly obtained from the machine data. On the other hand, the overlap interval and cross-sectional shape parameters should be measured from available microstructural analysis, e.g., SEM pictures (Figure 2) or other methods discussed in Section 4.4, as these are affected by other process parameters.

The geometric model depicted in Figure 25 and proposed by Angelo et al. [21], which is an extended version of that presented by Ahn et al. [23], works for any surface angle θ with only very minor deviations for $\theta \leq \theta_{s}$ as well as $\theta >\theta_{s}$. A comparison between the surface roughness obtained from the model and experiments showed good agreement, once more proving the model’s ability to realistically represent the cross-sectional shapes of FDM-printed parts.

**Table 2:** Printing process parameters negatively affecting the micromechanical composition.

Characteristic Mechanical Property	Printing Process Parameter
Strength	Raster orientation [2,6,7,8,15,34,50,51,52,53]
	Cross-sectional shape [6,8]
	Layer thickness [6,8]
	Overlap interval [6]
	Air gap [6,8]
	Porosity [5]
	Build direction [8]
	Bead width [8]
	Number of contours [8]
Fracture toughness	Build direction [29]
	Raster orientation [29,32,33]
Inter-layer bond strength	Cross-sectional shape [2,6,24,25,27,28,31,32,33]
	Printing temperature [31,32,33]
	Printing speed [31,32,33]
	Porosity [6]
	Raster orientation [15,50,51,52,53]
Stiffness	Porosity [7,9,28]
	Air gap [8,9,14,28]
	Raster orientation [2,7,8,14,28]
	Build direction [8,28]
	Cross-sectional shape [28]
	Layer thickness [8,28]
	Bead width [8,28]
	Overlap interval [28]
	Number of contours [8]

## 5. Conclusions

Several conclusions can be drawn from this review of the microstructural modeling of 3D-printed polymers. To begin with, the vast majority of experimental analyses of 3D-printed FDM parts are tensile tests performed on the specimen scale (ASTM dog bones). These experimental results are therefore not informative for smaller-scale (numerical) analyses, such as microstructural analysis. Furthermore, existing FEM analyses have mostly neglected the effects of internal bonds, i.e., the intra-layer and inter-layer bonds, with only a few recent exceptions. Rather, they have focused on the incorporation of porosity in geometrical representations of the material, but inter-layer and intra-layer bond properties like strength have rarely been considered. Only some recently published FEA studies using phase-field models for 3D-printed materials have actually distinguished between cross-layer or bulk layer failure (i.e., filament or bead failure) and inter-layer or inter-phase failure in their formulations. To the best of the authors’ knowledge, only one very recent multi-scale technique *explicitly* incorporated both the porosity and inter-facial bond properties (i.e., inter-layer and intra-layer bond strengths) simultaneously in a numerical (microstructural) model for 3D-printed polymers produced by material extrusion. Analytical models may be helpful in illustrating tensile failure strength and stiffness in the linear-elastic regime, but these are highly dependent on the numerous printing process parameters. On the other hand, microstructural and fractographic analyses can provide useful geometrical input. Resources like SEM pictures, microphotographs, optical microscopy images, and XCT can be used for determining cross-sectional shape parameters. Finally, geometrical analyses can be used to realistically represent and idealize the microstructural geometries of 3D-printed polymers.

From the conclusions above, it can be said that smaller-scale experiments are required to obtain appropriate input parameters, i.e., cross-sectional shape parameters, surface intact ratios, and intra-layer and inter-layer bond strengths. This allows for the explicit incorporation of internal bonds by not only considering porosity, but also including the (adhesive) bond strength between layers. Considering the significant influence of (small changes in) the printing process parameters on the characteristic mechanical properties of 3D-printed polymers, it is important to conduct tests with consistent printer settings at all length scales. When using consistent printing settings for all 3D-printed polymers, the geometrical input can be measured via microstructural analysis (e.g., XCT) to geometrically represent and idealize the respective microstructures using geometrical models.

However, capturing the (highly) anisotropic nature of FDM 3D-printed parts in microstructural models still requires additional attention. Before polymeric parts created through material extrusion are allowed to be used in final consumer products, it is important to develop robust numerical models that can adequately describe and predict their mechanical properties regardless of their shape and complexity. For instance, printing paths can be rather straightforward and parallel or significantly more complex when constantly changing direction within the same layer. Particularly in the latter case, it is important that constitutive relationships are assigned in accordance with their respective printing path orientations. Even though recent studies proposed for the first time promising multi-scale approaches that consider the cross-sectional shape of the microstructure including both the porosity and the inter-facial bond properties (i.e., inter-layer and intra-layer bond strengths) in numerical models for 3D-printed polymers produced by material extrusion, which is a major contribution to the field, these are still limited to parallel printing paths in each layer. When the printing paths are constantly changing direction (even within a single layer), the interface directions are also constantly changing, such that an additional level of direction-dependent anisotropy or *printing path-dependent anisotropy* is introduced.

In other words, there is a need for printing-path-dependent models that take microstructural information into consideration. The printing path orientations can be directly adopted from the (CAD) model’s G-code, while the assignment of printing-path-dependent properties can be achieved using a local coordinate system in which one of the axes is oriented in the direction of the printing path and the others correspond to the inter-layer and intra-layer bond phases, respectively. Such models will provide a more robust, realistic, and reliable modeling approach for 3D-printed polymers produced by material extrusion since they can capture the effects of both the inter-layer and intra-layer bonds as a result of this AM technique.

## Figures and Tables

**Table 1:** Summary of printing process parameters affecting characteristic mechanical properties.

Characteristic Mechanical Property	Printing Process Parameter
Tensile failure strength (TFS)	Raster orientation [18]
	Infill density [18]
	Bead width [46]
	Printing speed [46]
Inter-layer and intra-layer bonding	Raster orientation [6]
	Temperature (thermal history) [35]
Stiffness	Number of contours [8]
	Porosity [11]
Modulus of toughness	Infill density [5]
	Build direction [29]
	Raster orientation [29]
Deflection at failure	Infill density [28]
	Build direction [28]

## Abbreviations

The following abbreviations are used in this manuscript:

AM	Additive manufacturing
FDM	Fused deposition modeling
RP	Rapid prototyping
CAD	Computer-aided design
FFF	Fused filament fabrication
STL	Standard triangulation language
ABS	Acrylonitrile butadiene styrene
PLA	Poly-lactic acid
TPU	Thermoplastic polyUrethane
PC	Polycarbonate
PEI	Polyetherimide
PAEK	Polyaryletherketone
PEEK	Polyetheretherketone
SR	Solidity ratio
LWM	Low molecular weight
ML	Machine learning
TFS	Tensile failure strength
SFS	Shear failure strength
DCB	Double-cantilever beam
FEA	Finite element analysis
RVE	Representative volume element
XCT	X-ray computed tomography
UD	Unidirectional
ROM	Reduced-order model
FSDT	First-order shear deformation laminate theory
UC	Unit cell
TFA	Transformation field analysis
GT	Gurson–Tvergaard
SEM	Scanning electron microscope
